# Checkpoint Inhibition: Will Combination with Radiotherapy and Nanoparticle-Mediated Delivery Improve Efficacy?

**DOI:** 10.3390/medicines5040114

**Published:** 2018-10-23

**Authors:** Purushottam Lamichhane, Neha P. Amin, Manuj Agarwal, Narottam Lamichhane

**Affiliations:** 1 LECOM School of Dental Medicine, 4800 Lakewood Ranch Blvd, Bradenton, FL 34211, USA; plamichhane@lecom.edu; 2 Department of Radiation Oncology, University of Maryland School of Medicine, Baltimore, MD 21201, USA; Neha.Amin@umm.edu (N.P.A.); Manuj.Agarwal@umm.edu (M.A.)

**Keywords:** checkpoint inhibition, radiation therapy, resistance to therapy, biomarkers, combination therapy, liposomes, nanoparticles, PD-1, CTLA-4

## Abstract

Checkpoint inhibition (CPI) has been a rare success story in the field of cancer immunotherapy. Knowledge gleaned from preclinical studies and patients that do not respond to these therapies suggest that the presence of tumor-infiltrating lymphocytes and establishment of immunostimulatory conditions, prior to CPI treatment, are required for efficacy of CPI. To this end, radiation therapy (RT) has been shown to promote immunogenic cell-death-mediated tumor-antigen release, increase infiltration and cross-priming of T cells, and decreasing immunosuppressive milieu in the tumor microenvironment, hence allowing CPI to take effect. Preclinical and clinical studies evaluating the combination of RT with CPI have been shown to overcome the resistance to either therapy alone. Additionally, nanoparticle and liposome-mediated delivery of checkpoint inhibitors has been shown to overcome toxicities and improve therapeutic efficacy, providing a rationale for clinical investigations of nanoparticle, microparticle, and liposomal delivery of checkpoint inhibitors. In this review, we summarize the preclinical and clinical studies of combined RT and CPI therapies in various cancers, and review findings from studies that evaluated nanoparticle and liposomal delivery of checkpoint inhibitors for cancer treatments.

## 1. Introduction

The emergence of immunotherapy into the mainstream of oncology has been fueled by recent clinical advances and FDA approvals of inhibitors that block immune checkpoints in cancers, such as non-small-cell lung cancer, melanoma, head and neck squamous cell carcinoma, pancreatic ductal adenocarcinoma, lymphoma, and renal cell cancers [1,2,3]. Immune checkpoint molecules, such as programmed death receptor 1 (PD-1), programmed death-ligand 1 (PD-L1), cytotoxic T-lymphocyte-associated protein 4 (CTLA-4), T-cell immunoglobulin, and mucin domain-containing-3 (TIM-3), and lymphocyte-activation gene 3 (LAG-3), are expressed in various activated immune cells, tumor cells, and other tissues, and lead to inhibition of immune responses against tumors [4]. Two of the most studied checkpoint receptor/ligand interactions include PD-1/PD-L1 and CTLA-4/CD28. Intracellular signaling emanating from these interactions leads to suppression of effector immune responses against the tumors. Upon receptor/ligand interaction, the cytoplasmic tail of the receptor gets tyrosine phosphorylated on the tyrosine residue containing regions known as immunoreceptor tyrosine-based switch motifs (ITSMs) [5]. This leads to the recruitment of Src-homology domain-containing phosphatase 2 (SHP-2). The phosphatase activity of SHP-2 results in dephosphorylation of proximal signaling complexes, such as ZAP-70, PKC-θ, and PI3K, on T cells. This results in inhibition of proliferation, cytokine production, and cytotoxic activity of T cells, and promotes the apoptosis of T cells [5,6]. Studies recognized that immune-modulatory agents targeting these receptors have the potential to reinvigorate the antitumor immune responses, and that they represent an attractive modality for cancer therapy. Subsequent clinical studies of antibodies that block the interaction of these checkpoint molecules showed therapeutic responses leading to approvals in various cancers [1]. Unfortunately, the majority of the patients do not respond to these therapies [7], and identification of the determinants of responses remains scarce.

Preclinical and clinical studies have focused on understanding the determinants of success vs. failure of checkpoint inhibition (CPI) therapies. Among factors that determine failure include the innate barriers in the tumor microenvironment (TME), such as low infiltration of T cells, low expression of checkpoint receptors/ligands, and presence of immunosuppressive cells [7]. Therapy-induced adaptive resistance represents another determinant of CPI therapy failure. Such adaptive resistances include, but are not limited to, induction of immunosuppressive cytokines and additional checkpoint molecules, evolution of neoantigens, and mutations in β_2_-microglobulin and JAK1/2 [7,8,9,10]. In order to overcome these roadblocks in CPI therapies, combination therapies with radiation therapy (RT) are being studied in preclinical and clinical studies. Additionally, immune-related toxicities from systemic CPI therapies may prevent patients from obtaining the full clinical potential of these therapies. Immune dysfunction due to checkpoint molecules is often highest at the local tumor microenvironment, due to upregulated expression of checkpoint molecules on the infiltrating immune cells and the tumor tissues. While systemic CPI therapies target these checkpoint molecules, they also non-discriminately activate the systemic immune responses, leading to toxicities, and force the therapies to be halted before optimal responses are achieved. To overcome this barrier, studies are evaluating the feasibility and efficacy of targeted delivery of CPI, to the tumor microenvironment, using nanoparticles. Such studies are essential not only in understanding the mechanistic drivers of CPI therapy resistance, but also in devising and evaluating combination therapies that overcome such resistance and toxicities. The following sections will discuss the combination of RT with CPI in preclinical and clinical studies, and the latest advances in nanoparticle delivery of CPI.

## 2. Preclinical Studies of Radiation and Checkpoint Inhibitor Immunotherapy

Preclinical studies in various cancer models have demonstrated that the combination of RT and CPI therapy, such as anti-CTLA-4 and anti-PD-L1, can enhance antitumor immune responses and improve survival [11,12,13,14,15]. These preclinical studies, however, have not yet achieved a consensus on the best sequence of administration of these combination therapies or the RT dose and fractionation that would optimize therapeutic efficacies. There are many variables, both within the TME and surrounding stroma of tumors, that influence the results of these combination strategies. Hence, it is unlikely that there will be one standard treatment plan for all tumor types when combining CPI with RT (CPI-RT). The following section summarizes published preclinical studies that help build the knowledge and understanding of the complex interactions and effects of CPI-RT that could help informed design of future clinical trials.

When RT was combined with both PD-L1 and CTLA-4 blockade, further improvements in antitumor responses, complete responses (CRs), and survival proportions, were achieved in preclinical models. This synergy of anti-CTLA-4 or anti-PD-L1 with a range of RT doses and fractionations has been demonstrated in immune-competent mouse models of lung, breast, melanoma, and colorectal cancers [11,12,14,15,16,17,18]. Studies have shown that CPI enhances therapy responses in RT-resistant tumors that overexpress checkpoint molecules in response to RT. RT, on the other hand, improves antitumor responses in poorly immunogenic tumors that did not respond well to CPI therapies by enhancing T-cell receptor repertoire and immunogenic cell death, leading to tumor-antigen release [11,12,16,18]. In a mouse model of colorectal cancer (CT26 cell line), RT-mediated local control was significantly improved (*p* < 0.001), with concurrent anti-PD-L1 or anti-PD-1; resulting in curative rates of 66% and 86%, respectively [11]. Similarly, in an MC38 cell line model of colon cancer, the addition of RT to PD-L1 blockade significantly reduced tumor growth: RT vs. RT plus PD-L1 blockade = 278.6 ± 94.20 mm vs. 27.85 ± 27.85 mm (*p* = 0.034) [12]. In 4T1 breast cancer model, RT plus PD-L1 blockade significantly reduced tumor burden by 38% when compared to RT alone (RT plus PD-L1 vs. RT: 184.3 ± 13.5 mm^2^ vs. 292.8 ± 14.3 mm^2^, respectively; *p* < 0.01) and significantly improved survival (*p* < 0.001) [11]. Tumor growth was also significantly decreased with combination of RT and anti-PD-L1 in TUBO breast cancer mouse model (RT plus PD-L1 blockade vs. RT: 25.59 ± 10.26 mm vs. 402.8 ± 76.73 mm, *p* = 0.0002) [12]. When RT was combined with dual checkpoint blockade (anti-PD-L1 plus anti-CTLA-4), further improvements in complete responses (CRs) and survival were achieved in a preclinical model of breast cancer (RT plus anti-CTLA-4 resistant cell line). Survival was significantly increased (*p* = 0.014), and the CR rate was 56% for RT plus dual checkpoint blockade, compared to 33% for RT plus CTLA-4 blockade [18]. 

Additionally, RT induces an abscopal effect (antitumor responses outside the RT field) resulting in enhanced antitumor effects of CPI therapy [17]. When RT was combined with anti-PD1/PD-L1 therapy, both single and multiple fraction regimens (10–12 Gy × 1, 2 Gy × 5, and 4 Gy × 9 fractions) caused significant delays in tumor growth [11,12,19,20]. Similarly, a range of RT doses, combined with anti-CTLA-4, have led to reduced primary tumor growth of the irradiated tumor, including 12–20 Gy × 1, 12 Gy × 2, 8 Gy × 3, and 6 Gy × 5; however, only the fractionated regimens also led to abscopal effects [16,17,18]. Despite the encouraging outcomes of these studies, there was still no consensus on the ideal RT dose and fractionation, and researchers have turned to understanding the mechanism of RT-CPI synergy to drive their hypotheses and conclusions for design of optimal combination regimens. 

**Figure 1:**
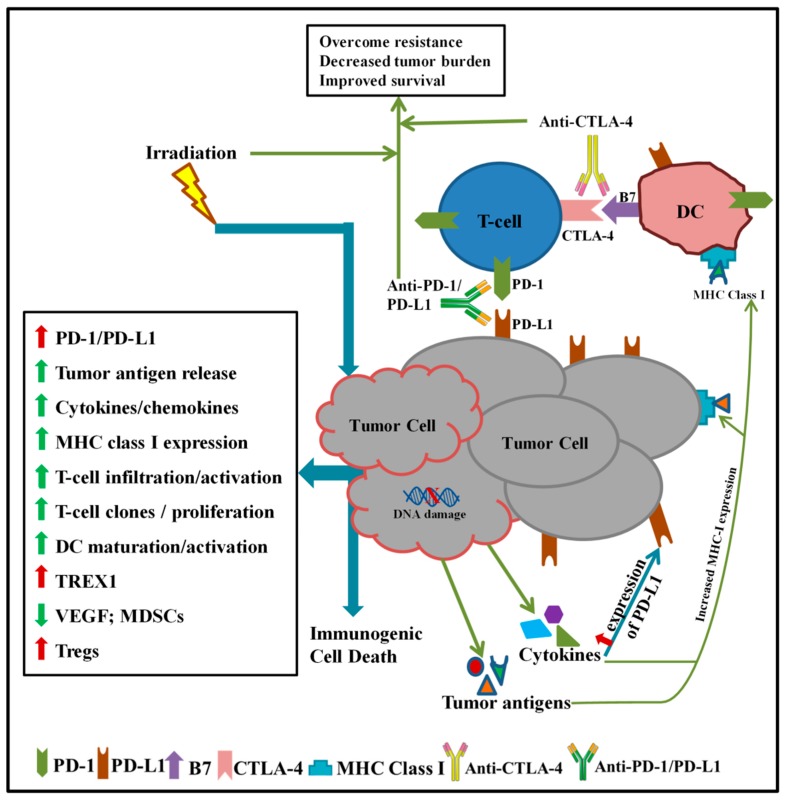
Schematic representation of radiation therapy (RT) and/or checkpoint inhibition (CPI) effects in the tumor microenvironment (TME).

The mechanism of synergy of RT-CPI has been described as RT acting as a booster or in situ vaccine to the TME immune system, resulting in delayed tumor growth with the addition of CPIs. RT causes double stranded DNA (dsDNA) breaks and subsequent tumor cell death, release of tumor antigens, increase in MHC class I expression, production of chemokines, and cell-adhesion molecules, increase in tumor infiltrating lymphocytes (TILs), and activation of T cells [21,22,23,24] (Figure 1). Upon interaction of irradiated tumor cells and dendritic cells (DCs), DCs acquire the DNA from the irradiated tumor cells. The cyclic GMP-AMP synthase (cGAS)-stimulator of interferon genes (STING) pathway then senses this cytoplasmic dsDNA, resulting in induction of interferon-β (IFN-β), a key mediator of dendritic cell maturation and cross-priming of CD8^+^ T cells [22,25]. Additionally, in response to the RT-induced pro-inflammatory milieu, PD-L1 and three prime repair exonuclease 1 (TREX1) can become upregulated in the TME, leading to attenuation of RT-induced immune responses and promotion of immunosuppression [11,12,22,26]. High levels of TREX1, in response to high-dose RT, leads to degradation of cytosolic DNA, hence preventing the cGAS-STING-dependent IFN-β production, DC activation, and subsequent cross-priming of CD8^+^ T cells [26]. Hence, appropriate RT doses and combination with CPI result in enhancement of antitumor responses while eliminating the roadblock presented by checkpoint molecules (Figure 1). 

It is also evident that the successes of RT-CPI treatments are dependent on a pre-existing immune response [19,27]; therefore RT-CPI may not be successful in patients without a pre-existing immunity. This was demonstrated in a preclinical study in which the inhibition of the tumor implantation-mediated development of tumor-resident antigen-specific T cells rendered mice unresponsive to RT-CPI [27]. In addition to polyclonal expansion of pre-existing T cells in the tumors, RT can also induce new clones of T cells to further stimulate antitumor immune responses and synergize with CPI [18,19,27]. Tumors that do not respond to CPI therapies often lack primed or pre-existing antigen-specific T cells [28]. Hence, therapy modalities, such as RT, that can expand and prime T cells in the TME, have the potential to derive enhanced antitumor responses and overcome resistance when combined with CPI [29]. 

RT and/or CPI can also improve therapy responses by modulating the immune-suppressive cells, such as myeloid-derived suppressor cells (MDSCs) and regulatory T cells (T_regs_), present in the TME. MDSCs are a heterogeneous population of immature cells of myeloid origin. These cells regulate immune responses by suppressing the T-cell responses. High dose (30 Gy) alone has been shown to decrease the frequency of MDSCs (unirradiated vs. irradiated = 26% vs. 6%) in tumors, while resulting in the increased infiltration of CD8^+^ T cells (unirradiated vs. irradiated = 19% vs. 70%); a process that was dependent on CD8^+^ antigen cross-presenting DCs, IFNγ secretion, and CD40L-expressing CD4^+^ T cells [30]. However, in this study, an extended fractionated regimen was found to be inefficient in controlling metastases or in enhancing survival. In another preclinical study, ablative hypofractionated RT, but not conventional fractionated RT, was shown to decrease the infiltration of MDSCs in the tumors through a mechanism dependent on inhibition of vascular endothelial growth factor (VEGF) production [20]. CTLA-4 and/or PD-1/PD-L1 blockade, in preclinical and clinical studies, has been shown to reduce MDSCs in the TME of various tumors [12,31,32,33,34]. The mechanisms of decreased MDSCs in these studies include decreased levels of CXCL1, inhibition of the CD47/SIRPα pathway, or antagonism by increased release of the T helper cell 1 (Th-1) type of cytokines. Combining RT with CPI (such as anti-PD-L1) can further decrease the MDSCs in TME (RT vs. RT plus anti-PD-L1 = 4.78% ± 2.49% vs. 0.38% ± 0.16% of total CD45^+^ cells), resulting in enhanced antitumor responses [12]. While RT reduces MDSCs, it increases the production and recruitment of regulatory T cells (T_regs_) in the TME [35]. CPI, on the other hand, decreases the infiltration of T_regs_ [33]. PD-1 blockade has been shown to decrease FoxP3 expression in a preclinical model [36], and T_regs_ from patients that responded to PD-1 blockade had diminished suppressive function [37]. While CTLA-4 blockade has been shown to selectively deplete T_regs_ in a Fc-Fcγ-dependent manner in mouse models [38], it is unable to deplete T_regs_ in human cancers [39], highlighting the need and potential for modifications of Fc portions for enhanced Fc-mediated depletion of T_regs_. RT-CPI decreases T_regs_ in the TME [40,41,42], suggesting that combined RT-CPI may overcome inefficacies of either treatment alone, by decreasing the immunosuppressive cells.

The effectiveness of RT and its combination with CPI may also depend on the dosing strategy. In a study, an RT dose of 11.5 Gy × 2 was able to inhibit VEGF receptor signaling, and lead to subsequent reduction in MDSCs, yet 4 Gy × 9 fractions did not have the same effect [20]. Also, while higher doses of RT can cause more dsDNA damage and promote cell death, RT doses above 12–18 Gy, per fraction, can also induce TREX1, which degrades cytoplasmic dsDNA, the component needed to trigger the STING pathway, resulting in decreased IFN-β and, subsequently, reduced abscopal effects. An RT dose between 8–12 Gy can result in the highest frequency of dsDNA breaks before triggering TREX1 elevation. Repeated doses of RT that do not trigger TREX1 would lead to increased IFN-β production, recruitment, and activation of dendritic cells, and subsequent priming of T cells for improved antitumor responses [26]. Therefore, multiple fractions of 8–12 Gy may be the ideal RT regimen to achieve a balance between immune-inhibitory and immunostimulatory signals in the TME for optimal antitumor responses [20,22,25,26]. Further studies, however, are necessary to determine the optimal dose(s) and fractionations that may convert each tumor type into an in situ vaccine for reliable antitumor responses.

In addition to the doses, timing and sequencing of RT and CPI are also crucial to the success of combined RT-CPI treatment. Combined RT-CPI has been shown to be effective if given concomitantly [11,19], but not sequentially (PD-1/PD-L1 blockade after RT) [11], with the efficacy of the concomitant treatment attributed to acute increase in PD-1 expression on the infiltrating T cells. The RT-induced surge in antigen presentation, TILs, and PD-1/PD-L1, only lasts for a couple days after RT; therefore, anti-PD-1/PD-L1 should be given ideally within 3–5 days of RT, if RT is the first treatment in the sequence [11,19]. Similarly, in a preclinical model of breast cancer, RT followed at least a day later by administration of anti-CTLA-4, improved survival compared to either treatment as monotherapy [16]. Pretreatment with anti-CTLA-4 followed by RT, however, was not evaluated. Alternatively, in a mouse model of colorectal cancer, treatment with anti-CTLA-4 one week prior to treatment with RT, was shown to be superior to treatment with anti-CTLA-4 one week after the RT [43]. This effect was attributed partly to the anti-CTLA-4-mediated depletion of T_regs_. Efficacy of stimulation of the co-stimulatory molecule OX40, however, relied on administration of agonist antibody at least a day after the RT [43]. Agonist OX40 antibody has also been shown to help overcome resistance to anti-PD-1/PD-L1 [44,45]. Anti-OX40 can boost tumor-specific T-cells in non-immunogenic mouse models prior to anti-PD-1/PD-L1 administration, resulting in improved local and distant tumor control and survival [43,44,45]. Timing of anti-OX40 is important to harness the efficacies of the combination treatments. If anti-OX40 is being used to enhance anti-PD-1/PD-L1 activity, it should be given several days prior to anti-PD-1/PD-L1 therapy [45]; however, if anti-OX40 is being used to enhance RT-effects, then it should be given immediately after RT to coincide with and harness the RT-induced antigen release and subsequent T-cell activation [43]. In the case of RT combination with a checkpoint blockade (CTLA-4) and checkpoint stimulation (OX40), Young et al. showed that administration of anti-CTLA-4 prior to RT, followed by OX40 stimulation, was the optimal combination in a mouse model of colorectal cancer [43]. These results highlight the complexities and need for further studies in determining the optimal sequencing of RT with inhibitors and agonists against different checkpoint and co-stimulatory molecules, respectively.

The last 20 years of preclinical research has helped to fill a void of understanding of the mechanisms of responses and resistances to combined RT-CPI in mouse models of cancers. Informed by such studies, various RT dosing and sequencing, in combination with CPI, are being evaluated in clinical trials. It will be interesting to see if these mechanisms, hypotheses, and expected outcomes are confirmed in these ongoing and future clinical trials with RT-CPI combination. 

## 3. Clinical Trials of Radiation and Checkpoint Inhibitor Immunotherapy

Ipilimumab (Bristol-Myers Squibb), a fully human CTLA-4 monoclonal antibody, was approved by the FDA in 2011 [46]. Monotherapy demonstrated improved long-term survival in subsets of patients with advanced melanoma [47,48]. An increased response rate has been demonstrated with 10 mg/kg, compared to lower dosing, without unacceptable toxicity [49], although non-response is still prevalent. Efforts have been made to enhance efficacy in a larger patient subset with the addition of other immune-modulating therapies. Ipilimumab, in combination with PD-1/PD-L1 blockade, has improved response rates and survival, albeit with increased toxicity and cost [49]. The application of immunotherapy has been broadened with data supporting use in cancers of the lung, kidney, and head and neck, with expansion of indications underway [50,51,52,53]. Table 1, Table 2 and Table 3, list the current clinical trials under investigation.

Preclinical, retrospective, and Phase 1 data using hypofractionated and/or stereotactic body radiation therapy (SBRT) combined with ipilimumab and/or PD-1/PD-L1 blockade have suggested synergism without added toxicity [54,55,56,57,58,59,60]. There is a suggestion that improved tumor responses may occur with higher radiation doses, and when delivered in close proximity to immunotherapy [61]. The current decade has witnessed an exponential increase in clinical research investigating combined immunotherapy and radiation therapy [62]. The earliest and most robust data have been presented for advanced melanoma, although there is emerging evidence in other solid and hematologic malignancies as well. The following section summarizes the results of completed and ongoing clinical trials evaluating the efficacy of RT-CPI on patients with various tumors.

### 3.1. Melanoma

The earliest prospective experience with combined CPI and RT was reported in 2015 from the University of Pennsylvania [18]. Twenty-two patients with metastatic melanoma were enrolled in a phase I trial in which a single index lesion received hypofractionated irradiation, followed by four cycles of ipilimumab. Among the 12 patients that were evaluated by PET for the irradiated lesion, none had progressive metabolic disease [18]. Of the unirradiated lesions, 18% of patients experienced a partial response (PR), 18% had stable disease (SD), and 64% had progressive disease (PD). Median progression-free survival (PFS) and overall survival (OS) was 3.8 and 10.7 months, respectively [18]. A subsequent phase I trial recruited 22 patients who received radiotherapy (both hypofractionated and standard fractionation) to one to two sites within 5 days of starting ipilimumab [55]. Fifty percent demonstrated clinical benefit, with 27.3% achieving ongoing complete response (CR) at median f/u of 55 weeks, and 27.3% achieving a PR for median of 40 weeks [55]. Patients who achieved a CR tended to have a smaller volume of disease and baseline, and experienced higher grade hypophysitis, in line with prior reports demonstrating improved control among patients who experience more significant immune-related toxicity [63].

Boutros et al. reported a phase 1 SBRT dose escalation trial in combination with ipilimumab (10 mg/kg for 4 doses) in 19 patients with advanced melanoma [54]. Radiotherapy was administered in 9, 15, 18, and 24 Gy in 3 fractions. Maximum tolerated dose (MTD) of 9 Gy was demonstrated, as two of six patients receiving 15 Gy experienced dose-limiting toxicity (DLT). The objective response rate (ORR) was 21%, with four patients experiencing PR and another four experiencing SD. The median PFS and OS were 7.2 and 4.4 months, respectively [54]. A similar trial was reported recently from Belgium [64]. Twelve patients with metastatic melanoma were enrolled in a phase 1 trial of dose-escalated SBRT (24 Gy, 30 Gy, and 36 Gy in 3 fractions) to one lesion and 4 cycles of ipilimumab at 3 mg/kg. SBRT was delivered before the third cycle of immunotherapy (IT). Local control was achieved in all but one irradiated patient, and the maximum tolerated dose (MTD) was not reached. Three patients experienced abscopal response in non-irradiated lesions. Grade 3–4 IT-related toxicity occurred in 25% of patients [64]. 

Given the high incidence of brain metastases in melanoma patients and poor intracranial response to ipilimumab alone, early combination experience with whole brain radiotherapy (WBRT) or stereotactic radiosurgery (SRS) has been reported to optimize intracranial control [65,66,67]. Efficacy and safety of combined SRS with PD-1 blockade was reported in retrospective single institution reports [68]. There is suggestion that the presence of radionecrosis is associated with prolonged OS and improved disease control [69]. Williams et al. reported a phase 1 trial of 16 patients treated with combined ipilimumab and either WBRT or SRS, depending on the degree of intracranial disease burden [70]. WBRT was delivered as 30 Gy in 10 fractions, and SRS was based on maximum tumor diameter or size of resection cavity, according to dose prescriptions on RTOG 90-05 trial [71]. Ipilimumab was started at 3 mg/kg on day 3 of WBRT, or 2 days after SRS, with an independent escalation of dose to 10 mg/kg. No patients experienced dose-limiting toxicity or radionecrosis. In contrast to the historical median of 4.7 months in melanoma patients with brain metastases, median OS was 8 months in the WBRT arm, and not reached in the SRS arm [72]. 

### 3.2. Central Nervous System

The efficacy of combined IT and RT for brain metastases is largely comprised of melanoma data, and has been described above. There is a lack of evidence supporting combination therapy for primary brain tumors. Keynote-028 demonstrated efficacy of pembrolizumab (anti-PD-1, Merck, Kenilworth, NJ, USA) in 26 PD-L1-positive recurrent glioblastoma-multiforme patients with a median OS of 14 months and median PFS of 3 months with a low rate of toxicity [73]. Multiple prospective trials are currently enrolling patients with high grade gliomas investigating the combination of RT and IT, such as CPI. 

### 3.3. Head and Neck

Patients who experience recurrence or metastases from a head and neck primary tumor often have a poor prognosis and limited therapeutic options. Nearly 40% of pathologic specimens demonstrate the presence of tumor infiltrating lymphocytes, providing a rationale for the efficacy of CPI [74]. Keynote-012 enrolled 60 patients in a phase Ib trial who received pembrolizumab at 10 mg/kg [74]. An overall response was seen in 18%, including 25% in HPV-positive patients and 14% in HPV-negative patients, and the drug was well tolerated [74]. Keynote-040 randomized 495 patients with recurrent squamous cell carcinoma of the oral cavity, oropharynx, hypopharynx, or larynx to pembrolizumab or investigator choice of standard doses of methotrexate, docetaxel, or cetuximab [53]. Although there was a higher overall response rate with pembrolizumab, there was no statistical difference in OS or PFS, albeit with lower grade (3–5) adverse events [53]. There is no current published prospective data on RT plus checkpoint blockade for the treatment of head and neck cancer.

### 3.4. Thoracic

#### 3.4.1. Non-Small Cell Lung Cancer (NSCLC)

Keynote-024 compared pembrolizumab vs. investigator’s choice of cytotoxic chemotherapy in 305 patients with an advanced NSCLC and PD-L1 tumor proportion score of ≥50% [75]. This phase 3 trial demonstrated the superiority of pembrolizumab, compared to platinum-based chemotherapy, with results showing an increased median PFS, increased overall survival at 6 months, and increased median duration of response with less treatment-related adverse events [75]. There is no prospective evidence supporting the addition of RT to anti-PD-1/PD-L1 therapy in advanced disease, with a number of ongoing trials. 

Thoracic SBRT regimens with biologically effective doses (BED) of approximately 100 Gy have been shown to have improve local disease control [76]. This dose is higher than that delivered with conventional radiation, and raises concerns about safety in combination with CPI. A phase 1 trial conducted at MD Anderson Cancer Center investigated concurrent or sequential SBRT to lung or liver lesions in a dose-escalated fashion combined with ipilimumab at 3 mg/kg [77]. Concurrent or sequential 50 Gy in 4 fractions or sequential 60 Gy in 10 fractions was prescribed to 35 patients. Response outside the radiation field, the primary response metric, demonstrated 10% partial response and 23% experienced clinical benefit (PR or SD lasting ≥6 months). Two patients receiving liver SBRT experienced DLT, one receiving 50 Gy concurrently, and the other receiving 50 Gy sequentially. There were no DLTs in the lung patients. Thirty-four percent experienced grade 3 toxicity, and no patients experienced grade 4–5 adverse effects [77]. A phase II study conducted in the Netherlands reported on 72 patients with advanced NSCLC randomized between of pembrolizumab alone or of pembrolizumab preceded by SBRT (8 Gy × 3 within 7 days) [78]. ORR was doubled (19% vs. 41%), and median PFS was tripled (1.8 vs. 6.4 months) with the addition of SBRT, demonstrating that SBRT augments the antitumor immune response [78].

#### 3.4.2. Small Cell Lung Cancer (SCLC)

SCLC is a highly aggressive malignancy, with 70% presenting with late stage disease. Nearly all patients experience local and/or distant progression, and no studied therapy to date has demonstrated an improvement over the standard of care (platinum-based chemotherapy) [79]. CheckMate 032 demonstrated durable efficacy and safety of nivolumab (anti-PD-1, Bristol-Myers Squibb) monotherapy, and in combination with ipilimumab, in a phase 1/2 trial [80]. A phase 3 trial evaluated the efficacy and safety of standard of care chemotherapy with or without ipilimumab in 954 patients with newly diagnosed extensive-stage small cell lung cancer [81]. Unfortunately, there was no difference in median OS or PFS with a higher rate of treatment-related discontinuation in the combination treatment group [81]. There are no reported prospective data investigating CPI with RT in SCLC. 

### 3.5. Breast

Breast cancer represents a spectrum of disease genotypes; among which, the triple negative and Her2-postive subtypes have been found to be immunogenic [82,83]. Early data on CPI monotherapy in locally advanced/metastatic breast cancer has demonstrated a modest benefit. Keynote-086 demonstrated an overall response rate of 5%, with median duration of response of 6.3 months in a subset of patients with heavily pretreated metastatic triple negative disease [84]. Ongoing studies are evaluating CPI in combination with standard cytotoxic chemotherapy. The addition of RT to pembrolizumab was assessed in a single arm phase II study of 17 patients, unselected for PD-L1 expression, with metastatic triple negative disease [85]. A dose of 30 Gy was delivered in 5 fractions of 6 Gy, within 3 days of pembrolizumab infusion. Of 9 evaluable women, 33% had PR, 11% SD, and 56% had PD with no added toxicities [85]. Several further trials are ongoing. 

### 3.6. Gastrointestinal

The role for CPI in locally advanced/metastatic esophageal, gastroesophageal junction (GEJ), and gastric cancers, is emerging. Keynote-028, a phase Ib trial, reported outcomes in 23 patients with advanced esophageal/GEJ tumors treated with pembrolizumab [86]. ORR was 30.4%, with 13% SD, and 12-month PFS of 21.7% with manageable toxicities [86]. Preliminary data from the phase Ib, Keynote-012 trial in 39 patients with gastric cancer treated with pembrolizumab, noted a 22% overall response, with a manageable toxicity profile [87]. There are no prospective data, to date, reporting on combined CPI and RT, although multiple trials are underway. 

There have been limited advances in the management of pancreatic adenocarcinoma. Patients typically present with locally advanced or metastatic disease. Concomitant chemoradiation extends median survival from 4.1 to 6.1 months, and gemcitabine administration adds another mere 1.24 months of survival. A single arm phase II study explored ipilimumab in 27 patients and demonstrated no response in all but one patient, who experienced delayed regression of the primary lesion and hepatic metastases [88]. To date, there have been no published prospective data on RT plus CPI for the treatment of pancreatic cancer. Similarly, there is limited data supporting the use of IT in hepatobiliary cancers or small/large bowel malignancies. 

### 3.7. Genitourinary

There are several treatments approved for the treatment of metastatic castrate resistant prostate cancer after progression with docetaxel chemotherapy, all of which have been demonstrated to improve OS compared to control [89]. Pathologic specimens often demonstrate inflammatory cell infiltrates, suggesting a host immune response. A phase I/II ipilimumab (10 mg/kg) dose escalation study, in combination with 8 Gy of RT to a bone lesion in 84 patients, reported efficacy with tolerable adverse effects [90]. Of 50 patients that received this dose, 8 had PSA declines of ≥50%, one had a CR, and six had SD [90]. CA184-043 was a multicenter phase 3 trial of men with metastatic castrate-resistant prostate cancer, who experienced progression after docetaxel chemotherapy [89]. A total of 799 patients received either bone-directed radiotherapy (8 Gy × 1) followed by ipilimumab (10 mg/kg) or placebo. Median OS was 11.2 vs. 10.0 months (*p* = 0.053), with an increase in toxicity among the patients receiving ipilimumab [89]. 

Renal cell carcinoma (RCC), that has disseminated, has limited therapeutic options that provide moderate overall survival benefit. Nivolumab was compared to everolimus in a phase 3 study of 821 pretreated patients with advanced clear-cell carcinoma, and demonstrated an OS of 25.0 vs. 19.6 months (*p* = 0.002), with a 25% ORR compared to 5% and lower grade 3–4 toxicities [51]. Ipilimumab was subsequently added to nivolumab, and compared to sunitinib in 1096 previously untreated patients [91]. The 18-month OS was 75% vs. 60%, and median survival was not reached, vs. 26.0 months. ORR was 42% vs. 27%, with lower grade 3–4 toxicities [91]. SBRT has been increasingly used in the management of inoperable primary RCC or management of metastatic disease, with overall local control of 85–100% [92]. There are no reported prospective data on combined IT/CPI and RT, and trials are currently underway. 

The use of intravesicular BCG in 1976 first demonstrated the efficacy of immunotherapy in urothelial carcinoma of the bladder [93]. Platinum-based chemotherapy, however, has been the standard of care for advanced diseases with limited overall survival benefit. A multicenter phase II trial reported on 310 patients with inoperable locally advanced or metastatic urothelial carcinoma, with progressive disease after platinum-based chemotherapy [94]. Patients received atezolizumab (anti-PD-L1, Roche, Indianapolis, IN, USA) and demonstrated an ORR of 26% with an ongoing response in 84% at median follow-up of 11.7 months, with 16% developing grade 3–4 adverse effects [94]. There are no reported prospective data of combined RT with CPI for urothelial carcinoma, and trials are underway. 

### 3.8. Gynecologic

Treatment options for recurrent and/or metastatic cancers of the cervix, uterus, vagina, and vulva, are limited after first-line therapy. CheckMate-358, a phase I/II study, reported preliminary results in 24 women with cancer of the cervix, vagina, and vulva, treated with nivolumab, and demonstrated an ORR of 20.8% and median PFS of 5.5 months [95]. Keynote-028 reported data on 28 women with locally advanced or metastatic PD-L1-positive endometrial cancer, with progressive disease after standard therapy. The patients were treated with pembrolizumab at 10 mg/kg every two weeks. Thirteen percent achieved PR, and 13% SD with median duration of response not reached, and with no patients experiencing grade 4 adverse events [96]. Although RT is commonly used in the management of primary and recurrent malignancies of the gynecologic tract, there are no reported data combining CPI with RT, and trials are ongoing.

## 4. Nanoparticle and Microparticle Delivery of Checkpoint Inhibitors

### 4.1. Nanoparticle Delivery of Checkpoint Inhibitors

The primary goal of cancer immunotherapy is to stimulate the host immune system to help eliminate cancer cells [97]. While CPI therapies embody this goal, they are often costly, delivered systemically, and may be discontinued in patients who have severe immune-related toxicities [98]. In this regard, nanoparticle delivery vehicles may overcome some of these barriers by improving stability and delivery of checkpoint inhibitors to tumor sites. Nanoparticles are particles that have a size in the range of nanometers. Different types of nanoparticles include, but are not limited to, liposomes, dendrimers, metal nanoparticles, carbon nanoparticles, silica nanoparticles, and magnetic nanoparticles. Various modifications of these nanoparticle platforms are often used to facilitate passive or targeted delivery of therapeutic and imaging agents to the tumor tissues. Design and composition of an ideal nanoparticle incorporates desired characteristics, such as biodegradability, ease of fabrication, cost-effectiveness, non-immunogenicity, and enhanced permeation and retention, with sustained release of payload at the tumor site [99]. Enhancing the therapeutic index of drug molecules is a major rationale of nanoparticle drug delivery systems, and modalities that incorporate CPIs with nanoparticle for therapies will not only have potential to improve therapeutic efficacies by enhanced delivery, but will also limit systemic toxicities [98]. In this regard, studies have explored the pharmacodynamics/pharmacokinetics, as well as therapeutic efficacies of CPIs’ incorporation into nanoparticle delivery vehicles for cancer therapies. 

#### 4.1.1. Polymeric and Metal Nanoparticle Delivery of Checkpoint Inhibitors

Metal-core nanoparticles and polymer nanoparticles have been studied for their efficacy in incorporating and delivering checkpoint inhibitors to tumor sites. A reporter polymeric nanoparticle, carrying paclitaxel, and which incorporated PD-L1-blocking antibodies through conjugation with PEG, showed enhanced antitumor activity in preclinical models of lung and breast cancer, leading to significantly decreased tumor volumes (*p* < 0.001) compared to control nanoparticles [100]. In another study, iron-dextran nanoparticles were conjugated with blocking antibody against PD-L1 and agonistic antibody against the co-stimulator 4-1BB [101]. This allowed for simultaneous blockade of checkpoint molecule, PD-L1, and stimulation of co-stimulatory molecule, 4-1BB, resulting in robust activation of tumor-infiltrating CD8^+^ T cells (increased CD107^+^ and IFNγ^+^ CD8^+^ T cells; *p* < 0.05), decreased average tumor size, and improved survival in preclinical models of melanoma and colon cancers. [101]. In melanoma model, the tumor sizes for antibody-conjugated nanoparticles (ACN) vs. no treatment were 112 mm^2^ vs. 205 mm^2^, respectively (*p* < 0.001) [101]. A significant decrease (*p* < 0.01) in tumor size was also observed with can, as compared to free antibody injections. Similarly, for colon cancer model, the tumor sizes were 19 mm^2^ vs. 158 mm^2^ (*p* < 0.001), for ACN vs. no treatment, respectively [101]. Animal survival in the colon cancer model was significantly increased from 10% for untreated mice to 70% (*p* < 0.001) for ACN-treated mice [101]. This study also determined that the in vivo half-life of ACN was 84.5 h, compared to 15.2 h for soluble antibody (*p* < 0.0001), with retention of 60% ACN as compared to 8% for soluble antibody at 72 h post-injection [101]. These studies highlight the potential for improved therapeutic efficacies and decreased toxicities, due to nanoparticle-mediated delivery of chemotherapeutic drugs, as well as immunomodulatory antibodies, such as checkpoint inhibitors, to the tumor site.

#### 4.1.2. Liposomal Delivery of Checkpoint Inhibitors 

Liposomes have been used as vehicles for chemotherapeutic drug delivery to the tumors. Liposomes are versatile nanoparticles that can be tailored for precision medicine. Multiple preclinical and clinical investigations, evaluating the use of nanoparticles and liposomes for delivering antibodies, genes etc. to the tumor sites, have emerged in recent years [98,102,103,104,105]. Liposomes are spherical lipid vesicles that are comprised of an aqueous core encapsulated by one or more lipid bilayers [106,107]. Modifications in preparation methods allow for generation of liposomal particles with different structures, colloidal size, surface charge, and chemical compositions as well as conjugations [106]. These design flexibilities can be exploited to create liposomes that can overcome barriers in drug delivery and imaging. In addition to chemotherapeutic drugs, modifications of liposomal delivery vehicles also permit attachment of different therapeutic and targeting antibodies, enabling targeted and sustained delivery to the tumor site. Characteristics of liposomes, such as biocompatibility, modulated pharmacokinetics, enhanced bioavailability, etc., make liposomes a promising delivery system for various drugs, genes, and immune therapies [106], and have led to preclinical and clinical investigations of the feasibility and efficacy of liposomes as therapeutic and diagnostic tools.

While liposomes have many advantages, one of the drawbacks of using conventional liposomes as drug carriers is their susceptibility to rapid clearance by the reticuloendothelial system. Scientists have tried to overcome this barrier by PEGylating the liposomes. PEGylation involves conjugation of the liposomal particles with polyethylene glycol (PEG). This increases size, and creates a protective hydrophilic layer on the surface of liposomes, resulting in decreased clearance by the reticuloendothelial system and kidneys [106,108]. The advantages of PEGylation include decreased immunogenicity, extended circulation time, enhanced pharmacokinetic profile, and improved drug solubility and stability [109]. In a recent study, efficacy of doxorubicin-loaded liposomes that were conjugated to DSPE-PEG-PD-1 monoclonal antibody, was evaluated. The results showed significant tumor growth inhibition (*p* < 0.05) with PD-1-conjugated liposomes compared to irrelevant IgG-conjugated liposomes [110]. These conjugated liposomes were also found to be stable for at least 48 h when incubated in serum, suggesting stability in biological systems [110]. A similar preclinical study with PEGylated liposomes carrying CTLA-4 blocking antibody showed improved accumulation into the tumor (PEGylated vs. non PEGylated vs. free anti-CTLA-4 = 7.57 + 1.55% ID/g vs. 0.63 + 0.43% ID/g vs. 1.06 + 0.42% ID/g respectively; *p* < 0.01; ID/g = injected dose per gram of tissue) and half-life, resulting in significant tumor growth delay (PEGylated vs. non PEGylated vs. free anti-CTLA-4 = 29.37% vs. −2.07% vs. 17.57% respectively) and improved median survival (PEGylated vs. non PEGylated vs. free anti-CTLA-4 = 34.98 vs. 22.27 vs. 30.12 days respectively; *p* = 0.0001) compared to non-PEGylated formulation or CTLA-4 antibody treatment alone [111]. Efficacy of PEGylated liposomes in delivering antibodies to the tumor site was confirmed in yet another study, with results also showing enhanced stability of liposomes and prolonged preservation of the secondary and tertiary structures of the delivered antibodies [112]. PEGylation of liposomes has been shown to reduce immunogenicity, and diminish complement activation and clearance by immune system [113,114], hence making them an attractive delivery vehicles for immunomodulatory antibodies, such as checkpoint inhibitors. Another liposomal formulation, nanohybrid liposomal cerasome nanoparticles (NLCNPs), was evaluated in a separate study [115]. Compared to non-conjugated PD-L1 administration along with paclitaxel, NLCNPs, carrying paclitaxel and conjugated with anti-PD-L1 antibodies, was significantly more efficient in delivering the drugs to the tumor site, resulting in enhanced tumor control and inhibition of metastases without added toxicities [115]. These studies underscore the benefit of using various formulations/modifications of liposomes for prolonged half-life and targeted delivery of checkpoint modulatory antibodies, without affecting their structure and function, to the tumor site for enhanced therapeutic efficacy.

### 4.2. Microparticles Delivery of Checkpoint Inhibitors

Silica and poly (lactic-co-hydroxymethyl-glycolic acid)-based microparticles have also been evaluated for delivery of CPIs and resulting therapeutic efficacies and toxicities [116,117]. Rahimian et al. showed that sustained release (up to 80% release in 30 days) of immunomodulatory antibodies at the tumor site, over time, can be achieved by intratumoral injection of antibodies loaded microparticles [116]. The microparticles were based on biodegradable poly (lactic-co-hydroxymethyl-glycolic acid) (pLHMGA), and were loaded with blocking antibody to CTLA-4, and agonistic antibody to CD40. Although the therapeutic efficacy of the antibody-loaded microparticles was similar to the control formulation (antibodies with incomplete Freund’s adjuvant (IFA)), significantly lower amounts of antibodies (5–10 times lower compared to antibodies in IFA) were detected in the serum of the microparticle formulation-treated animals, suggesting that this may lead to decreased systemic toxicities [116]. Similarly, in a mouse model of melanoma, intratumoral injection of functionalized mesoporous silica-based microparticles (with pore size up to 30 nm in diameter), that allowed for sustained release of anti-CTLA-4 antibody, slowed tumor growth (*p* < 0.05), and improved survival, compared to systemic administration of CTLA-4 blocking antibody or IgG conjugated microparticles [117]. Comparison with direct intratumoral injection of unconjugated anti-CTLA4 antibody, however, was not made in this study. These studies emphasize the potential for sustained release of CPIs at tumor sites, and decreased toxicities upon microparticle-mediated delivery.

While some clinical trials (such as NCT02158520 and NCT03107182) are evaluating the efficacy of nanoparticle delivery of chemotherapies in combination with systemic PD-1 or CTLA-4 blockade with or without RT, to the best of our knowledge, targeted deliveries of checkpoint inhibitors by nanoparticles or microparticles, with or without RT, have yet to be studied. It remains to be seen if the targeted delivery of CPIs using nanoparticles or microparticles enhances the therapeutic efficacy in combination with RT, and what schedule and dose combinations derive the best clinical outcomes.

## 5. Conclusions

Advances in clinical and preclinical sciences have shown that both CPI and RT have vast potentials in controlling and treating cancer malignancies. The clinical outcomes, however, are limited, due to innate or therapy-induced adaptive resistances that undermine the efficacy of RT or CPI as stand-alone treatments. Understanding the underlying mechanisms of resistance has led to studies aimed at evaluating the combination of RT and CPI. While results have been promising, these studies also highlight the need to further evaluate the sequence of treatments, doses, and fractionation schedules, and the type of checkpoint molecules targeted, in combination with RT, in order to generate the optimal therapeutic responses. Additionally, durability of the RT-CPI-generated T-cell responses, and determinants of abscopal responses, remains to be fully understood. Focused preclinical studies and ongoing clinical trials of RT-CPI should answer some of these outstanding questions, and aid in determination of optimal sequencing, dosing/fractionation, and selection of the RT-CPI treatments for specific tumor types.

One of the major roadblocks to successful CPI therapies against cancers includes the immune-related toxicities associated with systemic CPI treatments. In this regard, targeted delivery of checkpoint inhibitors has the potential to overcome this barrier. Various formulations of nanoparticles, liposomes, and microparticles, have been studied, to determine their feasibility as vehicles to deliver and provide sustained release of checkpoint molecules to the tumor site. Preclinical studies have shown that targeted delivery of checkpoint inhibitors not only enhances the efficacy of the treatments, but also decreases toxicities. While many clinical studies have evaluated antitumor efficacy of targeted delivery of chemotherapies and immunotherapies to tumor site, no clinical studies evaluating the nanoparticle/liposome/microparticle-mediated delivery of checkpoint molecules to TME are available. Additionally, nanoparticle- and microparticle-mediated delivery of CPI, in combination with RT, represents another opportunity to generate optimal antitumor responses with decreased toxicities. Further studies are warranted, however, to determine if such a combination has a clinical rationale.

## Figures and Tables

**Table 1:** Active Clinical Trials Combining CTLA-4 Blockade with Radiotherapy.

NCT Number	Phase	Title	Condition(s)	Systemic Therapy	Radiation Therapy	Outcome Measures
NCT01449279	2	A Pilot Study of Ipilimumab in Subjects with Stage IV Melanoma Receiving Palliative Radiation Therapy	Melanoma	Ipilimumab	RT to 1–2 sites	Primary: AEs Secondary: ORR, OS, duration of response
NCT03354962	1/2	Induction of Immune-mediated aBscOpal Effect thrOugh STEreotactic Radiation Therapy in Metastatic Melanoma Patients Treated by PD-1 + CTLA-4 Inhibitors (BOOSTER MELANOMA)	Melanoma	Nivolumab + ipilimumab	SBRT	Primary: DLT, abscopal effect, PFS Secondary: safety, PFS, pattern of response in irradiated vs. non-irradiated lesions.
NCT03601455	2	Phase II Study of Radiation Therapy and Anti-PD-L1 Checkpoint Inhibitor (Durvalumab) with or without Anti-CTLA-4 Inhibition (Tremelimumab) in Patients with Unresectable, Locally Advanced, or Metastatic Urothelial Bladder Cancer That Are Ineligible or Refusing Chemotherapy	Bladder Cancer Stage IVA-IVB	Arm 1: Durvalumab + EBRT Arm 2: Durvalumab + tremelimumab + EBRT	EBRT	Primary: AEs, PFS Secondary: LC, pCR, ORR, abscopal response, duration of response, OS
NCT02254772	1/2	A Phase I/II Study of Intratumoral Injection of SD-101, an Immunostimulatory CpG, and Intratumoral Injection of Ipilimumab, an Anti-CTLA-4 Monoclonal Antibody, in Combination with Local Radiation in Low-Grade B-Cell Lymphomas	Extranodal Marginal Zone B-Cell Lymphoma of Mucosa-Associated Lymphoid Tissue Nodal Marginal Zone B-Cell Lymphoma Recurrent Grade 1/2 Follicular Lymphoma Recurrent Marginal Zone Lymphoma Recurrent Small Lymphocytic Lymphoma Splenic Marginal Zone Lymphoma	TLR9 agonist SD-101 via intratumoral injections and ipilimumab via intratumoral injection + EBRT	Low dose RT to 1 site of disease	Primary: DLT Secondary: tumor response, TTP
NCT02115139	2	A Multicenter, Single Arm, Phase 2 Clinical Study on the Combination of Radiation Therapy and Ipilimumab, for the Treatment of Patients with Melanoma and Brain Metastases Actual Study Start Date: 4 April 2014 Actual Primary Completion Date: 31 December 2016 Estimated Study Completion Date: August 2018	Melanoma with Brain Metastases	Ipilimumab + RT	Whole-brain radiotherapy (WBRT) 30 Gy in 10 fractions	Primary: 1 year survival Secondary: PFS, PFS, OS, ORR, AEs
NCT02843165	2	Randomized Phase II Study of Checkpoint Blockade Immunotherapy Combined with Stereotactic Body Radiation Therapy in Advanced Metastatic Disease	Metastatic Cancer	Checkpoint blockade immunotherapy ± SBRT	SBRT: 28.5 Gy in 3 fractions of 9.5 Gy	Primary: ORR Secondary: safety/toxicity, PFS, OS, rate of stable disease, change in antitumor response
NCT02107755	2	A Phase 2 Study Using Stereotactic Ablative Radiation Therapy and Ipilimumab in Patients with Oligometastatic Melanoma	Liver Metastases Lung Metastases Recurrent/Metastatic Melanoma Melanoma Metastatic to Brain	Ipilimumab RT ipilimumab	Stereotactic radiosurgery	Primary: PFS Secondary: AEs, ORR, LF, OS
NCT03426657	2	First-Line Treatment of Locally Advanced HNSCC with Double Checkpoint Blockade and Radiotherapy Dependent on Intratumoral CD8^+^ T-Cell Infiltration	Locally Advanced Head and Neck Squamous Cell Carcinoma	Durvalumab + tremelimumab + RT	35 × 2.0/1.8/1.6 Gy	Primary: DLT Secondary: PFS, pCR, OS
NCT02701400	2	A Randomized Study of Tremelimumab Plus Durvalumab Combination with or without Radiation in Relapsed Small Cell Lung Cancer	Recurrent Small Cell Lung Carcinoma	Tremelimumab & durvalumab ± RT	SBRT	Primary: PFS, ORR Secondary: Immune-related objective response rate, OS
NCT01970527	2	RADVAX: A Stratified Phase II Dose Escalation Trial of Stereotactic Body Radiotherapy Followed by Ipilimumab in Metastatic Melanoma	Recurrent/Metastatic Melanoma	SBRT → ipilimumab	SBRT 3 fractions	Primary: Immune-related clinical response. Immune-related PFS, late toxicity, OS Secondary: Lymphocyte activation/analysis, T-cell response
NCT02888743	2	A Phase 2 Study of MEDI4736 (Durvalumab) and Tremelimumab Alone or in Combination with High- or Low-Dose Radiation in Metastatic Colorectal and NSCLC	Metastatic Non-Small Cell Lung Cancer Colorectal Cancer Stage IVA/IVB	Tremelimumab + durvalumab ± RT	High-dose daily RT Low-dose BID RT	Primary: ORR Secondary: PFS, PS, AEs, LC, abscopal responses
NCT03437200	2	Phase II Trial in Inoperable Esophageal Cancer Evaluating the Feasibility of the Combination of Definitive Chemoradiation with the Immune Checkpoint Blockers Nivolumab ± Ipilimumab	Inoperable Esophageal Cancer	Chemoradiation + nivolumab ± ipilimumab	RT: 50 Gy in 25 fractions of 2 Gy	Primary: PFS Secondary: Best overall response, pattern of progression, FFS, OS
NCT03522584	1/2	Recurrent/Metastatic Head and Neck Squamous Cell Carcinoma	Durvalumab (MEDI4376), Tremelimumab, and Palliative Hypofractionated Radiation (SBRT) in Patients with Recurrent/Metastatic Squamous Cell Carcinomas of the Head and Neck Previously Treated with Immune Checkpoint Inhibitors	Tremelimumab + durvalumab + SBRT	SBRT over 3 fractions	Primary: AEs Secondary: ORR, PFS, OS
NCT03604978	1/2	Grade II, III, or Recurrent Meningioma	A Phase I/II Study of Nivolumab Plus or Minus Ipilimumab in Combination with Multi-Fraction Stereotactic Radiosurgery for Recurrent High-Grade Radiation-Relapsed Meningioma	Nivolumab + radiosurgery ± ipilimumab	Multi-fraction stereotactic radiosurgery	Primary: MTD, AEs, ORR Secondary: PFS, OS, changes of peripheral T cells
NCT03604991	2/3	A Phase II/III Study of Peri-Operative Nivolumab and Ipilimumab in Patients with Locoregional Esophageal and Gastroesophageal Junction Adenocarcinoma	Adenocarcinoma of the Esophagus or Gastroesophageal Junction Stage I–IIIA	Arm 1: carboplatin, paclitaxel, radiation therapy Arm 2: carboplatin, paclitaxel, radiation therapy, nivolumab Arm 3: nivolumab Arm 4: nivolumab, ipilimumab	Radiation therapy once a week	Primary: pCR, DFS Secondary: AEs, OS, DFS
NCT03618134	Ib/II	Phase Ib/II Trial of Stereotactic Body Radiotherapy (SBRT) in Combination with Immunotherapy Prior to Transoral Robotic Surgery (TORS) for Human Papillomavirus Positive (HPV+) Squamous Cell Carcinoma of the Head and Neck (SCCHN)	HPV-Mediated (p16-Positive) Oropharyngeal Carcinoma Stages I–III	SBRT, durvalumab, TORS, neck dissection ± tremelimumab	SBRT in 5 fractions	Primary: AEs, PFS Secondary: OS, LC, DF, LRC
NCT02868632	1	A Phase I Study of Immune Checkpoint Inhibition (Anti-CTLA-4 and/or Anti-PD-L1) in Combination with Radiation Therapy in Patients with Unresectable and Non-Metastatic Pancreatic Cancer	Pancreatic Cancer	Arm1: MEDI4736 + SBRT Arm 2: Tremelimumab + SBRT Arm 3: MEDI4736 + Tremelimumab + SBRT	SBRT: 30 Gy in 5 fractions of 6 Gy	Primary: OS Secondary: PFS, response
NCT03275597	1	Comprehensive Stereotactic Body Radiotherapy (SBRT) to All Sites of Oligometastatic Non-Small Cell Lung Cancer (NSCLC) Combined with Durvalumab (MEDI4736) and Tremelimumab Dual Immune Checkpoint Inhibition	Metastatic Non-Small Cell Lung Cancer	SBRT followed by Durvalumab + tremelimumab	SBRT to all sites of disease. 30–50 Gy in 5 fractions	Primary: safety Secondary: PFS, OS
NCT03509584	1	Phase I Multicenter Trial Combining Nivolumab, Alone or with Ipilimumab, Plus Hypofractionated Radiotherapy for Pretreated Advanced Stage Non-Small Cell Lung Cancer Patients	Non-Small Cell Lung Cancer	RT + nivolumab ± ipilimumab	SBRT: 8 Gy × 3	
NCT01935921	1	A Phase Ib Trial of Concurrent Cetuximab (ERBITUX^®^) and Intensity Modulated Radiotherapy (IMRT) with Ipilimumab (YERVOY^®^) in Locally Advanced Head and Neck Cancer	Hypopharyngeal Squamous Cell Carcinoma Stage III–IVB Laryngeal Squamous Cell Carcinoma Stage III–IVB Oropharyngeal Squamous Cell Carcinoma Stage III–IVB (AJCC v7)	Cetuximab, RT, and ipilimumab	IMRT	Primary: DLT Secondary: clinical response, PFS, T-cell phenotypes, T regulatory cell counts, Myeloid-derived suppressor cell, HPV status
NCT03477864	1	R2810-ONC-16XX: A Phase 1 Neoadjuvant Study of Stereotactic Body Radiation Therapy with Systemic REGN2810 and Intraprostatic Ipilimumab, Alone or in Combination, in Patients with Locally Advanced Prostate Cancer Prior to Radical Prostatectomy	Prostate Cancer Stage II–IVB	Arm A: REGN2810, SBRT, surgery Arm B: ipilimumab, SBRT, surgery Arm C: REGN2810, ipilimumab, SBRT, surgery	SBRT for 4 fractions	Primary: AEs Secondary: pathologic response rate. PSA PFS, radiographic PFS
NCT03507699	1	Combination Treatment of Nivolumab, Ipilimumab, Intratumoral CMP-001 and Radiosurgery for Liver Metastases in Colorectal Carcinoma	Colorectal Cancer with Liver Metastases	Nivolumab +Ipilimumab + CMP-001 (TLR9 agonist) ± RT	SBRT: 21 Gy in three fractions to one liver metastasis	Primary: DLT Secondary: response rat, PFS
NCT01711515	1	A Phase I Trial of Sequential Ipilimumab After Chemoradiation for the Primary Treatment of Patients with Locally Advanced Cervical Cancer Stages IB2/IIA With Positive Para-Aortic Lymph Nodes Only and Stage IIB/IIIB/IVA with Positive Lymph Nodes	Cervical Cancer Stage IB–IVA	Cisplatin, radiation therapy, and ipilimumab	EBRT followed by intracavitary brachytherapy	Primary: DLT Secondary: Response rate, PFS, OS, location of recurrence, chronic toxicities

Compiled from www.clinicaltrials.gov. AEs: adverse effects, ORR: overall response rate, OS: overall survival; DLT: dose-limiting toxicity, PFS: progression-free survival, LC: local control, PCR: pathologic complete response, TTP: time to progression, FFS: failure-free survival, DFS: disease-free survival, SBRT (stereotactic body radiation therapy), EBRT (external beam radiation therapy), PSA (prostate specific antigen).

**Table 2:** Active Clinical Trials Combining PD-1/PD-L1 Blockade with Radiotherapy.

NCT Number	Phase	Title	Condition(s)	Systemic Therapy	Radiation Therapy	Outcome Measures
NCT03040999	3	Study of Pembrolizumab (MK-3475) or Placebo with Chemoradiation in Participants with Locally Advanced Head and Neck Squamous Cell Carcinoma (MK-3475-412/KEYNOTE-412)	Oropharyngeal Cancer (Independent of p16) Larynx/Hypopharynx Unresectable Oral Cavity Cancer	Arm 1: priming dose of Pembro before CRT. 2 cycles with RT along with 3 cycles of CDDP. 14 cycles of pembro maintenance Arm 2: placebo delivered at same schedule as pembro above	Accelerated or standard fractionation RT	Primary: EFS Secondary: OS, AEs, treatment discontinuations due to AEs, GHS/QoL, swallowing, speech and pain symptoms
NCT02992912	2	A Phase II Study to Assess the Efficacy of the Anti-PD-L1 Antibody Atezolizumab (MPDL3280A) Administered with Stereotactic Ablative Radiotherapy (SABR) in Patients with Metastatic Tumours	Metastatic Tumors	Atezolizumab 1200 mg every 3 weeks	Hypofractionated SABR: 45 Gy in 3 fractions of 15 Gy	PFS
NCT03115801	2	A Phase II Randomized Controlled Trial of Programmed Death-1/Programmed Death Ligand-1(PD-1/PD-L1) Axis Blockade Versus PD-1/PD-L1 Axis Blockade Plus Radiotherapy in Metastatic Genitourinary (Renal/Urothelial) Malignancies	Metastatic Renal Cell Carcinoma Metastatic Urothelial Carcinoma	Arm 1: Nivolumab or atezolizumab alone Arm 2: Nivolumab or atezolizumab + radiation	30 Gy in 3 fractions of 10 Gy	Primary outcome: best overall response rate Secondary: PFS, toxicity, OS
NCT03087864	2	PD-L1 Targeting in Resectable Oesophageal Cancer: A Phase II Feasibility Study of Atezolizumab and Chemoradiation	Resectable Esophageal Cancer Stages II–III	Carboplatin + paclitaxel + atezolizumab + radiation	23 fractions of 1.8 Gy	Primary: feasibility Secondary: toxicity, postoperative complications. Pathologic response, relationship between gut microbiota composition with response and toxicity
NCT03220854	2	Phase 2 Clinical Trial of Stereotactic Radiotherapy and PD-1 or PD-L1 Inhibiting Therapy for Treatment of Advanced Solid Tumors Progression on PD-1 or PD-L1 Inhibiting Therapy	Advanced Solid Tumors	Commercially available PD-1 or PD-L1 inhibitor + radiation	SBRT: 18–60 Gy in 3–5 fractions	Primary: OS, PFS per RECIST/RANO Secondary: OS, PFS per irRC
NCT02866747	1/2	A Phase I/II Multicenter Trial Evaluating the Association of Hypofractionated Stereotactic Radiation Therapy and the Anti-Programmed Death-Ligand 1 (PD-L1) Durvalumab (Medi4736) for Patients with Recurrent Glioblastoma (STERIMGLI)	Glioblastoma	Arm 1: hFSRT Arm 2: hFSRT + Durvalumab	24 Gy in 3 fractions of 8 Gy	Primary: dose-limiting toxicities, PFS Secondary: intracranial PFS, OS, safety/tolerability, QOL, neurologic/neurocognitive functions
NCT03474094	2	A European, Multicenter, Randomized, Open-label, Phase II Trial Aiming to Assess the Clinical and Biological Activity of an Anti-PD-L1 (Atezolizumab) in Operable Localized Soft Tissue Sarcomas Patients to be Treated with Radiotherapy	Soft Tissue Sarcoma	Arm 1: RT → atezolizumab → surgery Arm 2: Atezolizumab → surgery → RT Arm 3: RT → surgery → atezolizumab	50 Gy in 25 fractions of 2 Gy	Primary: pathologic response Secondary: PCR, at least 50% necrosis, % residual viable cells, ORR, tumor volume change, LRR at 1 year, TTR, DFS, immune cell infiltration, adverse events, amputation rates
NCT03446547	2	Ablative STEreotactic RadiOtherapy wIth Durvalumab (MEDI4736). An Open Label Randomized Phase II Trial with Durvalumab Following Stereotactic Body Radiotherapy (SBRT) in Patients with Stage I Non-Small Cell Lung Cancer (NSCLC)	Stage I NSCLC	Arm 1: SBRT Arm 2: SBRT → durvalumab		Primary: TTP Secondary: OS, LC, QoL, TTP by PD-L1 expression,
NCT03212469	1/2	A Phase I/II Study Evaluating the Safety and Clinical Activity of Anti-PD-L1 (Durvalumab [MEDI4736]) + Anti CTLA-4 (Tremelimumab) Antibodies Administrated in Combination with Stereotactic Body Radiotherapy (SBRT) in Patients with Metastatic Squamous Cell Carcinoma of Head and Neck, Lung, Oesophagus, Cervix, Vagina, Vulva, or Anus	Head and Neck Squamous Cell Carcinoma, Lung Cancer, Esophageal Cancer	Durvalumab + tremelimumab + SBRT at C1D15 → Durvalumab		Primary: DLT
NCT03421652	2	Phase II Trial of Concurrent Nivolumab in Urothelial Bladder Cancer with Radiation Therapy in Localized/Locally Advanced Disease for Chemotherapy Ineligible Patients [NUTRA]	Stage II–IV Bladder Urothelial Carcinoma	Nivolumab + RT	Radiation therapy on weeks 1, 3, 5, 7, and 9.	Primary: PFS Secondary: adverse events, ORR, MFS, OS, QOL, PD-1, and PD-L1 expression, Th1/Th2 cytokine ratio
NCT02311361	1/2	A Pilot Study of Immune Checkpoint Inhibition (Durvalumab with or without Tremelimumab) in Combination with Radiation Therapy in Patients with Unresectable Pancreatic Cancer	Pancreatic Cancer	Tremelimumab/durvalum or both + RT	SBRT: 8 Gy × 1 of 5 Gy × 5	Primary: safety Secondary: plasma pharmacokinetic, OS, ORR, PFS
NCT02968940	2	A Phase II, Open-Label, Single Arm, Multicenter Study of Avelumab with Hypofractionated Radiation in Adult Subjects with Transformed IDH Mutant Glioblastoma	Glioblastoma	Avelumab 10 mg/kg every 2 weeks + RT	30 Gy in 5 fractions of 6 Gy	Primary: safety, PFS Secondary: OS, median PFS, ORR, duration of response
NCT02913417	1/2	A Feasibility Study of Sequential Hepatic Internal Radiation and Systemic Ipilimumab and Nivolumab in Patients with Uveal Melanoma Metastatic to Liver	Uveal Melanoma	Yttrium 90 + ipilimumab 3 mg/kg every 3 weeks × 4 + nivolumab 1 mg/kg every 3 weeks × 4 then nivolumab 3 mg/kg every 2 weeks until progression or 3 years	SIR-Spheres Yttrium 90	Primary: safety/tolerability Secondary: clinical efficacy, immunologic changes, correlation of tissue markers and response to immunotherapy, tumor melanin
NCT03407144	2	An Open-label, Uncontrolled, Multicenter Phase II Trial of MK-3475 (Pembrolizumab) in Children and Young Adults with Newly Diagnosed Classical Hodgkin Lymphoma with Inadequate (Slow Early) Response to Frontline Chemotherapy (KEYNOTE 667)	Hodgkin Lymphoma	Arm 1: ABVD (doxorubicin, bleomycin, vinblastine and dacarbazine) induction pembrolizumab + AVD chemotherapy (doxorubicin, vinblastine, dacarbazine) × 2 followed by RT Arm 2: OEPA (vincristine, etoposide/etopophos, prednisone/prednisolone and doxorubicin) induction pembrolizumab + COPDAC-28 chemotherapy (cyclophosphamide, vincristine, prednisone/prednisolone, dacarbazine) × 4 RT if PET response	21 Gy with boosts to 30 Gy for PET-avid sites	Primary: ORR Secondary: rate of negative PET, EFS, OS, frequency of RT, AE
NCT03116529	1/2	Neoadjuvant Anti-PD-L1 (Durvalumab/MEDI4736) Plus Anti-CTLA-4 (Tremelimumab) and Radiation for High Risk Soft-Tissue Sarcoma	Soft Tissue sarcoma	Durvalumab 1500 mg + tremelimumab 75 mg every 4 weeks × 3 concurrent with RT followed by surgery followed by maintainence Durvalumab until disease progression	50 Gy in 25–28 fractions of 1.8–2.0 Gy/fraction. Tumors > 10 cm receive a single 15 Gy fraction of high-dose spatially fractionated (GRID) radiation therapy within 1–3 days prior to radiation therapy	Primary: toxicity, pathologic response Secondary: OS, DSS, RFS, radiologic response
NCT02530502	1/2	Phase I/II Trial of Radiation Therapy Plus Temozolomide with MK-3475 in Patients with Newly Diagnosed Glioblastoma (GBM) Study Start Date: October 2015 Estimated Primary Completion Date: March 2019 Estimated Study Completion Date: November 2020	Glioblastoma	RT with concurrent temozolomide + pembrolizumab followed by temozolomide and pembrolizumab × 6 or until disease progression or unacceptable toxicities	focal RT	Primary: MTD Secondary: PFS Tertiary: PD-1/PD-L1 expression and T-cell infiltration. Correlate MGMT status with outcome
NCT03469713	2	Nivolumab Plus Stereotactic Body Radiotherapy (SBRT) in II and III Line of Patients with Metastatic Renal Cell Carcinoma (mRCC)	Metastatic Renal Cancer	Nivolumab + RT followed by nivolumab for responders until PD or toxicities	30 Gy in 3 fractions of 10 Gy to a metastatic disease site	Primary: ORR Secondary: PFS, OS, ORR of irradiated and non-irradiated metastases, AEs
NCT03283943	1	Phase I (Safety Assessment) of Durvalumab (MEDI4736) with Focal Sensitizing Radiotherapy in Platinum Resistant Ovarian, Primary Peritoneal or Fallopian Tube Epithelial Carcinoma	Ovarian Cancer, Primary Peritoneal Carcinoma, Fallopian Tube Cancer	Durvalumab + RT	Focal sensitizing radiotherapy: Starting dose level of 24 Gy in 4 fractions of 6 Gy and may be escalated to 32 Gy in 4 fractions of 8 Gy	Primary: MTD Secondary: ORR, Ca-125 response rate, immune-related response rate
NCT02400814	1	Pilot Study of MPDL3280A Plus Stereotactic Ablative Radiotherapy (SAR) in Stage IV Non-Small Cell Lung Cancer	Stage IV Non-Small Cell Lung Cancer	Arm 1: Concurrent MPDL3280A (anti-PD-L1, every 3 weeks) + SBRT Arm 2: MPDL3280A followed by concurrent SBRT starting on 3rd course Arm 3: SBRT followed by MPDL3280A	SBRT	Primary: AEs, response rate using irRECIST, PFS
NCT02837263	1	Pembrolizumab in Combination with Stereotactic Body Radiotherapy for Liver Metastatic Colorectal Cancer	Colorectal Cancer Stage IVA/IVB	SBRT followed by single cycle of pre-operative pembrolizumab followed by surgery to remove all known sites of metastatic disease; followed by pembrolizumab alone	SBRT 40–60 Gy in 5 fractions	Primary: 1 year recurrence rate Secondary: time to recurrence, DFS, OS
NCT02735239	1/2	Phase 1/2 Study of Anti-PD-L1 in Combination with Chemo (Radio)Therapy for Oesophageal Cancer	Oesophageal Cancer	Arm 1: Durvalumab + standard of care chemotherapy Arm 2: Durvalumab + tremelimumab + standard of care chemotherapy Arm 3: Recommended combination of doses from Cohort A1 or A2 Arm 4: durvalumab, + surgery + standard of care chemotherapy Arm 5: Durvalumab + surgery + standard of care chemotherapy + radiotherapy		Primary: AEs, dose-limiting toxicity, change in baseline laboratory evaluations Secondary: Tumor response, PFS, OS, 1 year survival
NCT02621398	1	Moving PD-1 Blockade with Pembrolizumab into Concurrent Chemoradiation for Locally Advanced Non-Small Cell Lung Cancer	Non-Small Cell Lung Cancer Stages II–IIIB	Paclitaxel + carboplatin + pembrolizumab + RT	3DCRT or IMRT	Primary: MTD and DLT Secondary: ORR, MFS, OS, PFS
NCT02608385	1	Phase I Study of PD-1 Blockade by Pembrolizumab With Stereotactic Body Radiotherapy in Advanced Solid Tumors	Solid Tumors	RT followed by Pembrolizumab	SBRT	Primary: recommended SBRT dose Secondary: AEs, response rate, PFS, OS, LC
NCT02444741	1/2	Phase I/II Trial of MK-3475 and Hypofractionated Stereotactic Radiation Therapy in Patients with Non-Small Cell Lung Cancer (NSCLC)	Lung Cancer	RT + Pembrolizumab	SBRT) to a total dose of 50 Gy in 12.5 Gy fractions (4 fractions total). Wide-field radiation therapy (WFRT) delivered at 45 Gy in 15 daily fractions	Primary: MTD Secondary: PFS
NCT02696993	1/2	Phase I/II Trial of Nivolumab with Radiation or Nivolumab and Ipilimumab with Radiation for the Treatment of Intracranial Metastases from Non-Small Cell Lung Cancer	Metastatic Brain Cancer	Nivolumab +RT ± ipilimumab	SRS: physician prescribed dose; WBRT: 30 Gy in 10 fractions	Primary: recommended dose Secondary: intracranial PFS, neurocognitive changes
NCT03050554	1/2	Phase I/II Study of the Safety, Tolerability, and Efficacy of Stereotactic Body Radiation Therapy (SBRT) Combined with Concurrent and Adjuvant Avelumab for Definitive Management of Early Stage Non-Small Cell Lung Cancer (NSCLC)	Early Stage Non-Small Cell Lung Cancer	Avelumab + RT	SBRT: 12 Gy × 4 fractions or 10 Gy × 5 fractions (4–5 radiation doses given over 10–12 days every other day)	Primary: safety/tolerability Secondary: LRC, OS
NCT02658097	2	A Phase II Trial of Pembrolizumab Sequentially Following Single Fraction Non-Ablative Radiation to One of the Target Lesions, in Previously Treated Patients with Stage IV NSCLC	Stage IV Non-Small Cell Lung Cancer	Pembrolizumab ± RT	8 Gy × 1 fraction	Primary: RECIST response Secondary: PFS, PS, LC
NCT02434081	2	A Phase II Trial Evaluating the Safety and Efficacy of the Addition of Concurrent Anti-PD-1 Nivolumab to Standard First-Line Chemotherapy and Radiotherapy in Locally Advanced Stage IIIA/B Non-Small Cell Lung Carcinoma	Non-small Cell Lung Cancer Stage III	Nivolumab concurrent with standard chemoradiotherapy	EBRT	Primary: ≥grade 3 pneumonitis Secondary: PFS, time to pneumonitis, ORR, TTF
NCT02831933	2	ENSIGN: Phase II Window of Opportunity Trial of Stereotactic Body Radiation Therapy and In Situ Gene Therapy Followed by Nivolumab in Metastatic Squamous or Non-Squamous Non-Small Cell Lung Carcinoma and Metastatic Uveal Melanoma	Lung Cancer	Nivolumab + ADV/HSV-tk intratumoral injection + Valacyclovir + RT	30 gray (Gy; 6 Gy × 5 fractions)	ORR, OS, PFS, AEs

Compiled from www.clinicaltrials.gov. EFS: event-free survival, GHS: global health score, QoL: quality of life, MFS: metastasis-free survival, TTR: time to relapse, FFS: failure-free survival, irRC (immune-related response criteria), hFSRT (hypofractionated stereotactic radiation therapy), →: followed by, TTF (time to treatment failure).

**Table 3:** Active Clinical Trials Combining OX40 Stimulation with Radiotherapy.

NCT Number	Phase	Title	Condition(s)	Systemic Therapy	Radiation Therapy	Outcome Measures
NCT01862900	1/2	Phase I/II Study of Stereotactic Body Radiation Therapy to Metastatic Lesions in the Liver or Lung in Combination with Monoclonal Antibody to OX40 (MEDI6469) in Patients with Progressive Metastatic Breast Cancer After Systemic Therapy	Breast Cancer Metastatic to Lung/Liver	SBRT → MEDI6469	SBRT: Cohort 1: 15 Gy (central tumors 10 Gy) Cohort 2: 20 Gy (central tumors 15 Gy) Cohort 3: 20 Gy × 2 (central tumors 15 Gy × 2).	Primary: DLT Secondary: response rate in both irradiated and non-irradiated tumors
NCT01303705	1/2	Phase Ib Study of Monoclonal Antibody to OX40, Cyclophosphamide (CTX) and Radiation in Patients with Progressive Metastatic Prostate Cancer After Systemic Therapy	Metastatic Prostate Cancer	Anti-OX40 **+** cyclophosphamide (300 mg, 600 mg, or 900 mg) + RT	8.0 Gy in 1 fraction to a maximum of three bone metastatic deposits	Primary: MTD Secondary: immune and clinical responses
NCT03410901	1	Intratumoral Injection of SD-101, an Immunostimulatory CpG, in Combination with BMS-986178 and Local Radiation in Low-Grade B-Cell Lymphomas	Follicular Lymphoma Grade 1–3a Lymphoplasmacytic Lymphoma Mantle Cell Lymphoma Marginal Zone Lymphoma Small Lymphocytic Lymphoma	Radiation therapy + SD-101 + BMS-986178	Radiation therapy on days 1–2	Primary: DLT Secondary: ORR, PFS

Compiled from www.clinicaltrials.gov.

## Abbreviations

PD-1	programmed death receptor 1
PD-L1	programmed death-ligand 1
CTLA-4	cytotoxic T-lymphocyte-associated protein 4
CPI	checkpoint inhibition
TME	tumor microenvironment
RT	radiation therapy
AEs	adverse effects
ORR	overall response rate
OS	overall survival
DLT	dose-limiting toxicity
PFS	progression-free survival
LC	local control
pCR	pathologic complete response
TTP	time to progression
FFS	failure-free survival
DFS	disease-free survival
SBRT	stereotactic body radiation therapy
EFS	event-free survival
GHS	global health score
QoL	quality of life
MFS	metastasis-free survival
TTR	time to relapse
